# Israeli necropolitics and the pursuit of health justice in Palestine

**DOI:** 10.1136/bmjgh-2023-014942

**Published:** 2024-02-01

**Authors:** Layth Hanbali, Edwin Jit Leung Kwong, Amy Neilson, James Smith, Sali Hafez, Rasha Khoury

**Affiliations:** 1 Institute of Community and Public Health, Birzeit University, Birzeit, State of Palestine; 2 Independent Researcher, Berlin, Germany; 3 University of Sydney, Sydney, NSW, Australia; 4 Department of Epidemiology and Public Health, Institute of Epidemiology and Health Care, University College London, London, UK; 5 Department of Health Services Research and Policy, Faculty of Public Health and Policy, London School of Hygiene & Tropical Medicine, London, UK; 6 Department of Obstetrics & Gynaecology, Division of Fetal Medicine and Complex Family Planning, Boston University School of Medicine, Boston, Massachusetts, USA

**Keywords:** Health policy, Health systems, Public Health, Global Health

**Summary box**

- We abhor the continuation and acceleration of the Israeli state's systemic violence against the Palestinian people. We reassert that resolution of the settler colonial root causes of violence in Palestine is central to the pursuit of justice and peace.
- The moral foundations of global health and medical practice require us to prioritise and foreground oppressed realities, and to practise epistemic resistance.
- Framing Palestinian violence on October 7 as provocation and Israeli violence as response is ahistoric and indicates indifference to the everyday violence experienced by Palestinians. The Israeli state practises both fast violence against Palestinians, while simultaneously creating the conditions for their ‘slow death’.
- The systematic targeting and destruction of the health system and healthcare workers in Gaza has been central to Israel's military strategy, while many Israeli officials have expressed clear genocidal intent.
- The occupation of Palestine demonstrates the horrors of Israeli necropolitics, which leads to the creation of 'death-worlds' in which people survive and resist in perpetual proximity to death.

In the 10 weeks since our *BMJ Global Health* editorial was published,^1^ a further 16 882 Palestinians have been killed as a result of Israel’s ongoing violence in Gaza, the West Bank and East Jerusalem.^2 3^ As of 28 December, at least 21 624 people are known to have been killed across occupied Palestine since 7 October, with thousands of people still missing, many of whom are presumably trapped under destroyed buildings.^3^ More than 59 618 people in Gaza and the West Bank have been injured.^3^ At least 1.9 million people—over 85% of the population—are now internally displaced across Gaza.^4 5^

The horrific scale of Israel’s latest attacks validates the concerns and calls raised in our editorial: namely that Israel’s ongoing military violence in Gaza is an extension of the longstanding, systemic violence intrinsic to the Israeli state’s colonisation and occupation of Palestine. Connections can be clearly traced between the exploitation and dispossession of people, land and resources that defined European colonial violence, ongoing neocolonial exploitation worldwide, and every aspect of Israel’s settler colonial violence in Palestine today.^6^ We reaffirm our unwavering commitment to actions that expose and challenge sites of exploitative and extractive power and violence. People’s health, lives and freedoms are at risk.

We acknowledge the many complementary and critical responses to our editorial, including on the journal’s rapid response platform and Greenland *et al*’s commentary.^7^ However, we recognise that demands for ‘corrections’ are almost always demands to acquiesce to Israeli state propaganda and its hasbara strategy, which seek to promote a blinkered defence of the Israeli state and manipulate and distort the full extent of its violent intent and violent actions.^8^ We reject these demands since they represent a potent form of censorship that seeks to elevate the narratives of those who wield colonial and other forms of power while suppressing the voices of Indigenous, marginalised and oppressed people. This, in turn, contributes to knowledge erasure and epistemic violence, following from which we must take seriously the moral responsibility to practise epistemic resistance.^9^

With this in mind, we engage with five themes that predominate in the responses to our editorial. First, alleged omissions and accusations of one-sidedness. We do not hesitate to foreground the lives, narratives and rights of Palestinian people. To do so with intentionality is an attempt to redress the cumulative neglect, misrepresentation and obfuscation of the experiences of Palestinians. Such accusations are typical of efforts to undermine solidarity and justice movements, wherein those wielding hegemonic power attempt to distort our understanding of the targets, perpetrators and root causes of oppression and injustice. This, in turn, undermines our ability to express and enact meaningful forms of solidarity. Our commitment to health, equity and justice requires that we stand with all oppressed people globally. This in no way negates other injustices.

Second, the suggestion that Israel only chose violence in Gaza as a result of Hamas’ attacks on 7 October. This claim is patently false. Recent evidence that exposes this falsehood includes Israel’s escalation of attacks in the Israeli-occupied and partially Palestinian authority-administered, West Bank,^10^ the incarceration of Palestinian citizens of Israel based on their social media activity,^11^ and the systemic torture of Palestinian political prisoners.^12^ This violence is multifaceted and has a long history. Even the most cursory review of the killings, home demolitions and displacement perpetrated by Israeli soldiers and settlers between 1948 and September 2023 is indicative of the protracted nature of Israel’s strategy of violence and attempted subjugation of the Palestinian people.

Claiming that the violence only started on 7 October indicates acceptance of, or indifference towards, the everyday violence and inhumanity experienced by the Palestinian people. Attempts to dehistoricise and decontextualise the present encourage us to ignore the many ways in which the Israeli state dictates both life and death for the Palestinian people, either through the fast violence of aerial bombardments, or what Berlant referred to as ‘slow death’^13^: visible in the progressive dispossession of Palestinians who are crammed into ever-shrinking spaces, the denial of life-sustaining necessities and services, the destruction of livelihoods, repeated physical assaults and disablement, mass incarceration, extensive restrictions on movement (including to seek healthcare), and now ethnic cleansing in Gaza executed by mustering Palestinians through a dystopian grid of ever-shifting, supposedly ‘safe zones.^14^

Third, the suggestion that the Israeli response to the attacks on 7 October is justified. These suggestions are horrific. Nothing can justify the use of advanced military technologies to indiscriminately kill an equivalent of one Palestinian every 5 minutes for 2 months, the displacement of at least 1.9 million people, or the bombardment of Gazans as they attempt to seek safety. Current and former Israeli government officials have not only attempted to justify this violence, but some have advocated ethnic cleansing and genocide against the Palestinian people.^15^ To cite a small number of these instances, a prominent Israeli think tank stated that the invasion of Gaza presented a ‘rare opportunity’ to remove all Palestinians from Gaza and into Egypt.^16^ In keeping with Israel’s strategy of collective punishment, an Israeli minister suggested ‘there are no non-combatants in Gaza’, and described a nuclear attack on Gaza as ‘an option’.^17^ A member of the Israeli Knesset has called for a ‘Nakba that will overshadow the Nakba of 48’,^18^ while another Israeli minister has called for a ‘Gaza Nakba 2023’.^19^ An Israeli minister has been tasked with developing a plan to ‘thin the population in Gaza to a minimum’,^20^ and more recently, the head of a local authority in Israel has called for the entirety of Gaza to be ‘emptied and levelled flat, just like in Auschwitz’.^21^ With respect to Israeli military actions and statements of political intent, there can be no doubt that we are bearing witness in real time to the ‘eliminatory settler colonial strategies’ that we described in our earlier editorial.^22^

Fourth, the suggestion that Israeli attacks on healthcare are necessary or justified. Again, such suggestions are horrific and should be roundly condemned, particularly by medical and public health professionals. Despite claims made by the Israeli military that armed groups have used health facilities for military purposes, according to Human Rights Watch no evidence has yet been presented that provides legal justification for the loss of the protected status of hospitals and other health infrastructure, as codified by International Humanitarian Law.^23^ An extensive Washington Post investigation of the Israeli attacks on, and invasion of, Al-Shifa Hospital found that the evidence provided by Israel does not demonstrate that the hospital was being used as a command and control centre, nor that the hospital building is connected to a larger tunnel network.^24^ Notably, the burden of proof remains with the attacker to provide adequate evidence that the protected status of a health facility has been lost; ‘in case of doubt, there should be a presumption of civilian status’.^25^ If a health facility loses its protected status, the principles of proportionality, precaution and distinction still apply.^25^

Despite these supposed protections, according to Dr Ghassan Abu Sitta, an experienced conflict surgeon who provided care in Gaza between 9 October and 19 November, the ‘destruction of the health system has been the main thrust of the (Israeli) military strategy’.^26^ As investigative journalists and forensic analysts continue to scrutinise the provenance of the Al-Ahli Hospital munitions strike on 17 October, the Israeli military has proceeded to commit hundreds of attacks on protected health services and personnel across Gaza and the West Bank. The WHO has recorded a horrifying 238 attacks on healthcare in Gaza alone between 7 October and 14 December.^27^ In this brief period, 26 of 36 hospitals in Gaza have been damaged. Only 13 remain partially functional as a result of Israeli bombardments, ground attacks and military raids, coupled with the effects of Israel’s acute-siege-on-chronic-blockade and the manufactured scarcity of life-sustaining resources.^3 27^

Patients, staff and displaced people have been forcibly displaced from hospitals, health workers have been detained,^28^ and besieged people have been shot at as they have attempted to flee.^29^ At least 340 Palestinian healthcare workers have been killed by the Israeli military,^30^ while at least 52 Palestinian healthcare workers have been abducted, of whom 40 remain in detention.^31^ Many of these healthcare workers were killed or detained while on duty.

Fifth, the suggestion that Israel is not colonising Palestine. This position ignores the well-documented reality of the ongoing domination, exploitation and dispossession of the Palestinian people, and the historical violence and mass displacement that enabled the formation of the state of Israel in 1948. It also ignores the historical accounts of Zionists themselves, whose self-stated aspirations make clear that the settlement of Palestine was and is a colonial and imperial project.^32 33^ The biopower and necropower that Israel exercises over Palestinian lives in their own land demonstrates the extent of its colonial grip.^34^

The many forms of violence—from the physical to the epistemic—enacted by the Israeli state all cause illness and death. The recognition of the systematic nature of this violence, and the pervasiveness of Israeli state control over almost every aspect of the everyday lives of Palestinians, made the philosopher Achille Mbembé declare that: ‘The most accomplished form of necropower is the contemporary colonial occupation of Palestine’.^35^ It is the power to dictate the terms of life and death, and ultimately who lives and who dies. Repeatedly framing Palestinian violence as a provocation and Israeli violence as a response is a product of ignorance to the necropower exercised by the Israeli state. Necropower and necropolitics are enabled in places that Achille Mbembé termed ‘death-worlds’, where ‘vast populations are subjected to conditions of life’ that enable a precarious form of survival in perpetual proximity to death.^35^ Within this world, there is gross indifference to Palestinian suffering and extreme obfuscation of the horrors of Israeli necropolitics.

As health professionals, and moreover, as concerned global citizens, we share a duty to bear witness and to act to redress injustices wherever they manifest. We reject the suggestion that medicine and public health can, or should be, apolitical. Rather, we understand Israeli colonial dispossession and control, its occupation, its apartheid system, and its extreme and ongoing genocidal violence as leading determinants of health, life, and death in Palestine.^36^ To this end, it is incumbent on us to reaffirm the global demands for an immediate ceasefire to end the genocidal violence enacted in Gaza, an immediate arms embargo to prevent further violence, wide-reaching and immediate action to safeguard human rights for all people in the region, the investigation and prosecution of all war crimes, and most importantly the meaningful and sustained pursuit of justice and the right to self-determination for the Palestinian people.

## Data Availability

All data cited in this paper is publicly available.
